# A sustainable bioprocess to produce bacterial cellulose (BC) using waste streams from wine distilleries and the biodiesel industry: evaluation of BC for adsorption of phenolic compounds, dyes and metals

**DOI:** 10.1186/s13068-024-02488-3

**Published:** 2024-03-12

**Authors:** Erminta Tsouko, Sotirios Pilafidis, Konstantina Kourmentza, Helena I. Gomes, Giannis Sarris, Panagiota Koralli, Aristeidis Papagiannopoulos, Stergios Pispas, Dimitris Sarris

**Affiliations:** 1 Theoretical and Physical Chemistry Institute, National Hellenic Research Foundation, 48 Vassileos Constantinou Ave., 11635 Athens, Greece; 2 Laboratory of Physico-Chemical & Biotechnological Valorization of Food By-Products, Department of Food Science & Nutrition, School of Environment, University of the Aegean, Leoforos Dimokratias 66, 81400 Lemnos, Greece; 3 Food, Water, Waste Research Group, Department of Chemical & Environmental Engineering, Faculty of Engineering, University of Nottingham, University Park, Nottingham, NG7 2RD UK; 4 Green Chemicals Beacon of Excellence, University of Nottingham, University Park, Nottingham, NG7 2RD UK; 5 Institute of Chemical Biology, National Hellenic Research Foundation, 11635 Athens, Greece

**Keywords:** Wine industry, Low-cost feedstock, BC-based adsorbent, Heavy metals, Dephenolization, Wastewater treatment

## Abstract

**Background:**

The main challenge for large-scale production of bacterial cellulose (BC) includes high production costs interlinked with raw materials, and low production rates. The valorization of renewable nutrient sources could improve the economic effectiveness of BC fermentation while their direct bioconversion into sustainable biopolymers addresses environmental pollution and/or resource depletion challenges. Herein a green bioprocess was developed to produce BC in high amounts with the rather unexplored bacterial strain *Komagataeibacter rhaeticus,* using waste streams such as wine distillery effluents (WDE) and biodiesel-derived glycerol. Also, BC was evaluated as a bio-adsorbent for phenolics, dyes and metals removal to enlarge its market diversification.

**Results:**

BC production was significantly affected by the WDE mixing ratio (0–100%), glycerol concentration (20–45 g/L), type of glycerol and media-sterilization method. A maximum BC concentration of 9.0 g/L, with a productivity of 0.90 g/L/day and a water holding capacity of 60.1 g water/g dry BC, was achieved at 100% WDE and ≈30 g/L crude glycerol. BC samples showed typical cellulose vibration bands and average fiber diameters between 37.2 and 89.6 nm. The BC capacity to dephenolize WDE and adsorb phenolics during fermentation reached respectively, up to 50.7% and 26.96 mg gallic acid equivalents/g dry BC (*in-situ* process). The produced BC was also investigated for dye and metal removal. The highest removal of dye acid yellow 17 (54.3%) was recorded when 5% of BC was applied as the bio-adsorbent. Experiments performed in a multi-metal synthetic wastewater showed that BC could remove up to 96% of Zn and 97% of Cd.

**Conclusions:**

This work demonstrated a low-carbon approach to produce low-cost, green and biodegradable BC-based bio-adsorbents, without any chemical modification. Their potential in wastewater-treatment-applications was highlighted, promoting closed-loop systems within the circular economy era. This study may serve as an orientation for future research towards competitive or targeted adsorption technologies for wastewater treatment or resources recovery.

## Background

The direct use of agro-industrial waste and food residues to produce sustainable biopolymers and other bioeconomy-oriented products addresses major modern issues related to the deterioration of newly extracted resources, as well as management of waste biomass and effluents that are conventionally disposed in aquatic and/or terrestrial ecosystems, landfilled or incinerated [1]. Distilleries are the second most water-intensive industry after the pulp and paper industry. It is reported that around 12–15 L aqueous waste, the so-called vinasse or distillery stillage or distillery wastewater, is generated per one L of ethanol produced [2]. Wine distillery effluents (WDEs) are generated from wine distilleries, as leftovers of grape pomace upon ethanol distillation. WDEs are high-risk pollutants due to their low pH, high biochemical and chemical oxygen demand, high solids concentration, and considerable presence of phenolic compounds such as benzoic acids, hydroxycinnamic acids, and flavonoids. These make their disposal, management and alternative valorization routes challenging [3]. WDEs are conventionally disposed in ponds, or they are used as soil fertilizers since they are rich in nitrogen, phosphate and potassium compounds. However, extensive usage results in severe water and ground pollution [4]. In Spain, alcohol-producing companies have to pay to the municipalities 230 euros/month/m^3^ for the treatment of vinasse from distilleries, plus any extra fees, based on the type and amount of the alcohol produced. Moreover, fines for the unauthorized discharge of vinasses to a river course can reach up to 40,000 euros [5]. Crude glycerol is produced as a co-product from the industries of soap and fatty acids production, while the traditional transesterification pathway to produce biodiesel generates crude glycerol at a volumetric ratio of 10%. Glycerol is a low-cost material due to surplus production (around 7.66 × 10^6^ tons each year), cost-intensive steps that are required for purification (to remove residual catalyst, methanol, fatty acids, soaps, and salts), and the limited market capacity to absorb its growing production [6, 7].

Access to safe water is the most basic human need. Despite this, advances in manufacturing of agricultural products, textiles, paints, and pharmaceuticals have led to the contamination of aquatic territories with dyes, phenolic compounds, solvents, and heavy metals (most commonly Cd, Cr, Cu, Ni, and Zn). Their removal is extremely important due to severe adverse effects in human and animal health, as well as the environment. Common chemical precipitation generate toxic sludge [8] while adsorption technology especially when natural, biobased and multifunctional polymers are used could offer cost-efficiency, simplicity, high removal rates and variety of available adsorbents (organic, inorganic and biobased) [9, 10]. Lately, biosorption approaches using renewable and sustainable materials such as lignin, banana and pomegranate peels, activated carbon from agro-residues, agricultural waste from shells, fibers, husks, straw, etc. have been developed [11].

Bacterial cellulose (BC) is produced with high efficiency from bacteria of the genera *Komagateibacter.* BC presents superior properties to plant-derived polymers while it is devoid of pectin, hemicellulose, lignin and minerals. BC has exceptional physicochemical profile and barriers to gases and thus it is very promising for novel applications in the food industry, biopolymer industry, the biomedical sector, pharmaceuticals and environmental bioremediation [12, 13]. BC could be a promising bio-adsorbent due to its specific properties. More specifically, BC is composed of a highly porous 3-D fibrillar network that provides high surface area and thus active sites for adsorption. The interconnected pores within the BC network create pathways for the diffusion of contaminants into the material, enhancing adsorption kinetics. Also due to its inherent hydrophilicity BC interact strongly with polar waterborne contaminants including ions, polar organic molecules, and hydrophilic dyes, facilitating their adsorption from aqueous solutions. BC possesses mechanical robustness and so it can withstand repeated regeneration cycles without significant loss of its adsorption capacity. BC is biocompatible and it can be properly functionalized or modified to preferentially adsorb certain pollutants or valuable metals/molecules contained in waste streams/effluents such as WDE or biodiesel-derived glycerol [14]. Zhou et al. [4] investigated several natural and renewable materials e.g., chitosan, sugarcane bagasse, alginates, rice straw, crop residues and BC as adsorbents for tetracycline. BC showed significantly higher adsorption capacity (up to 118 mg/g) of tetracycline compared to the aforementioned materials probably due to increased porosity of BC enabling more surface area available for tetracycline adsorption. The authors highlighted the BC potential use as a functional biomaterial in the adsorption technology.

The biosynthesis of BC is catalyzed by multi-enzymes including glucokinase, phosphoglucomutase, UDP-glucose phosphorylase, and cellulose synthase with uridine diphosphoglucose being the primary sugar nucleotide precursor. When the fermentation media contains hexoses and three-carbon compounds (e.g., glycerol, dihydroxyacetone, pyruvate) BC biogenesis follows the pentose phosphate pathway (PPP) [16]. Alternatively, dihydroxyacetone, pyruvate, and carboxylic acids can enter the metabolic route of gluconeogenesis combined with the tricarboxylic acid cycle (TCA) to be converted into BC. bcs operons are drivers in the BC pathway since they are responsible for the intracellular formation of cellulosic ribbons, the extracellular transport of cellulosic microfibrils through the bacterial membranes, and the self-organization of cellulosic fibrils into highly structured clusters [17].

In this study, BC production was investigated via a one-factor-at-a-time approach, considering the effect of different mixing ratios of WDE, the crude glycerol concentration, type of glycerol and sterilization method of the fermentation media on BC production. The water holding capacity (WHC), surface morphology (SEM), and functional groups (FTIR) of BC samples were monitored. The capacity of the produced BC to dephenolize WDE and absorb phenolic compounds was *in-situ* examined. BC was also investigated as a bio-absorbent of dyes (acid yellow 17) and metals including Mn, Co, Ni, and Cd.

The main novelty of this study was the development of a sustainable approach for the direct valorization of toxic waste streams that derive from the biodiesel industry and wine distilleries, to produce BC in substantial amounts. The capacity of BC as a bio-adsorbent of phenolics, metals and dyes was also investigated to enlarge its pool of applications including wastewater treatment approaches.

## Methods

### Raw materials

Crude glycerol was kindly provided by the biofuels industry Elin Verd SA (Greece). Crude glycerol was decanted in a separation funnel for 48 h prior to use to reach a purity of 85% w/w. WDE was supplied from a local distillery of Greek spirits (Greece), as the liquid fraction of grape pomace leftovers after the red wine distillation. WDE was filtered (muslin cloth), and centrifuged (10.595 RCF, 4 °C, 10 min, Hettich Universal-320R, Germany). The supernatant was stored at –20 °C for further use.

### Microbial strain, and pre-culture conditions

The bacterial strain *Komagataeibacter rhaeticus* UNIWA AAK2 was isolated from Kombucha beverage, and it was kindly provided by Dr Maria Dimopoulou (Department of Wine, School of Food Science, University of West Attica). BC production (pH value and C/FAN ratio), as well as maintenance of the strain and pre-culture preparation were performed according to Tsouko et al. [18]. Briefly, media for inoculum preparation constituted of 20 g/L glucose, 5 g/L peptone, 5 g/L yeast extract, 2.7 g/L Na_2_HPO_4_, 1.15 g/L citric acid while the pH was adjusted to 6 using 4 M NaOH. Pre-cultures were incubated for 2 days, at 30 °C and 180 rpm.

### BC production

The evaluation of BC production was performed based on the one-factor-at-a time methodology. Initial shake flask fermentations took place using WDE solutions of 0%, 25%, 50%, 75% and 100% v/v, diluted with tap water. The fermentation media was supplemented with crude glycerol to reach ≈20 g/L of total initial glycerol concentration. Experiments were also performed using different glycerol concentrations (ca. 30 g/L and 45 g/L) while the nitrogen amount was kept stable (ca. 300 mg/L free amino nitrogen-FAN). A fermentation was also carried out using ≈30 g/L pure glycerol and WDE based on optimal result of the previous investigation. Up to this phase of experiments, the fermentation media was thermally sterilized at 120 °C for 15 min (Raypa autoclave) to achieve sterile conditions. Additional experiments were carried out with fermentation media being filter-sterilised (0.2 μm filter unit, Polycap™ AS, Whatman Ltd.).

All fermentations were carried out in a batch mode in 250 mL Erlenmeyer flasks (50 mL working volume, static conditions) at 30 °C using 10% (v/v) inoculum. Yeast extract (5 g/L) and peptone (5 g/L) were used as nitrogen sources. The fermentation broth was supplemented with 2.7 g/L Na_2_HPO_4_ and 1.15 g/L citric acid while the pH was maintained at 6 during fermentation using 4 M NaOH [19]. The glycerol concentration and the FAN consumption, along with the dry BC weight, were monitored at specified time intervals. All experiments lasted 11 days.

### Downstream of BC and properties determination

BC membranes were removed from the fermentation media and immersed in 0.5 M NaOH, followed by 30 min of heating at 90 °C. Subsequently, the BC membranes were repeatedly rinsed with distilled water until a pH value of 6–6.5 was obtained and they were oven-dried at 35 °C until a constant weight was reached (48 h). WHC of BC membranes was determined as reported by Efthymiou et al. [20]. Briefly, the membranes were shaken to remove excess surface water, weighed, dried. The WHC was calculated as the mass of water removed divided by the dry weight of BC. The surface morphology of the BC specimens was examined using a Schottky Field Emission ultra‐high resolution Scanning Electron Microscope (FE‐SEM, JEOL 7610F, Japan). The samples were sputtered with Pd/Au using a Quorum SC7620 sputter/coater (Quorum Technologies Ltd, UK). Imaging was carried out at a maximum operating range of 10 kV, and the surface micrographs were taken at increasing magnifications and at a scale of 1 µm. ImageJ software was used to analyze fiber diameters. Attenuated total reflectance Fourier Transform infrared spectroscopy (ATR-FTIR) was carried out on a Bruker Equinox 55 instrument equipped with an attenuated total reflectance diamond accessory from SENS-IR and a press. 64 scans (500–5000 cm−1) at 2 cm^−1^ resolution were performed (for BC and dry WDE samples).

### Adsorption of phenolics compounds and dephenolization

Herein the potential of the microbial strain to dephenolize the WDE that was used as the fermentation media (in different percentages) was evaluated. More specifically adsorption experiments of crude phenolic compounds were performed as described by Liu et al. [21] with slight modifications. Briefly, crude phenolic compounds were recovered from the fermentation media (at 0 h and after 10 d of fermentation via liquid–liquid extraction using ethyl acetate at a ratio of 3:1 v/v (30 min, mixing every 10 min). After phase separation, the upper phase was collected, and the total phenolic content (TPC) was determined based on the Folin–Ciocalteu colorimetric method as reported by Tsouko et al. [22]. Briefly, aliquots of the collected phase were incubated with Folin-Ciocalteu reagent (0.2 N) and Na_2_CO_3_ (75 g/L) (90 min, 30 °C). Absorbance was measured at 765 nm using a double-beam UV–Vis spectrophotometer (Shimadzu, UV-1900i). The concentration of TPC was expressed in mg of gallic acid equivalent (GAE) per L of fermentation media). The substrate dephenolization capacity (%) was calculated using Eq. (1).

1 $$\%\, Substrate\, dephenolization =\frac{{TPC}_{0}-{TPC}_{f}}{{TPC}_{0}}\times 100$$

where TPC_0_ represents TPC of the fermentation media at the beginning of fermentation (0 h), and TPC_f_ is the TPC of fermentation media after 10 days of fermentation. The absorption of crude phenolic compounds in the BC membranes was calculated using Eq. (2).

2 $$\%\, Phenolics\, absorption=\frac{{TPC}_{0}-{TPC}_{f}}{{BC}_{dry}}$$

where BC_dry_ represents the dry weight of BC (in g/L) produced at the end of each fermentation.

### Dye removal

A dye solution of 2 mL (20 mg/L of acid yellow 17, CAS 6359-98-4) [3 replicates] was mixed with different concentrations of BC (0.5, 1, 2, and 5 wt.%) in Eppendorf tubes. The solution was mixed using a Benchmark Scientific Rotor mini shaker at 30 rpm for 30 min. Subsequently, tubes were centrifuged at 12,200 rpm for 5 min (Eppendorf mini spin ultracentrifuge). Absorbance was measured at 404 nm as λ_max_ using a UV/Vis spectrophotometer (Agilent Cary 60 UV–Vis).

### Metal removal

A multi-metal synthetic wastewater was prepared by dissolving 0.2 g MnO_4_Cl_2_∙4H_2_O, 0.16 g CoCl_2_∙6H_2_O, 0.60 g NiCl_2_∙6H_2_O, 0.4 g ZnCl_2_, and 0.02 g CdSO_4_ in one L of ultrapure water (MilliQ 18.2 MΩ cm). The solution pH was adjusted from 3 to 7 with 5 M NaOH. The metal removal from the solution was investigated in batch tests (triplicates), in conical flasks (250 mL) containing 1 g of BC and 100 mL of synthetic wastewater (at 20 °C, 150 rpm). Aqueous samples (1 mL) were taken after 5, 10, 15, 25, 40, 60, 80, 100, and 120 min, filtered through a syringe filter (0.22 µm, mixed cellulose, Kinesis) and diluted in 2% HNO_3_ (Romil SpA™ Super Purity Acid) for ICP-MS analysis. Control experiments without BC were conducted to assess metal removal through mechanisms other than adsorption (e.g., precipitation or solution chemistry changes). The removal of the dye and metals was calculated using Eq. (3).

3 $$\%\, Removal =\frac{{C}_{0}-{C}_{f}}{{C}_{0}}\times 100$$

where C_0_ represents the initial concentration, and C_f_ is the final concentration after treatment with BC.

### Analytical methods

The concentrations of glucose, fructose and glycerol were determined using a High-Performance Liquid Chromatography (HPLC). The HPLC system was equipped with a refractive index detector (RID-10A; Shimadzu Corp., Japan), column oven (CTO-10ASvp; Shimadzu Corp., Japan), and a ReproGel H column (250 × 8 mm, 9 μm; Dr. Maisch, Germany). The mobile phase was a 5 mM H_2_SO_4_ aqueous solution with 0.6 mL/min flow rate at 40 °C oven temperature [23].

The chemical structure of pure and crude glycerol was determined using ^1^H-NMR spectroscopy. The ^1^H-NMR experiments were conducted on a Varian 300 MHz spectrometer (Palo Alto, CA, USA). The samples (0.1 mL) were dissolved in 0.6 mL of deuterated oxide (D_2_O) [24].

Multi-element analysis of diluted solutions was undertaken by ICP-MS (Thermo-Fisher Scientific iCAP-Q; Thermo Fisher Scientific, Bremen, Germany). Internal standards typically included combinations of Sc (10 µg/L), Ge (10 µg/L), Rh (5 µg/L), Re (5 µg/L), and Ir (5 µgL), and were used to correct for instrumental drift, being introduced to the sample stream on a separate line (equal flow rate) via the ASXpress unit or added directly to calibration standards and samples and introduced on a single line. The matrices used for internal standards, calibration standards and sample diluents were 2%. Primary grade HNO_3_ with 4% methanol was used to enhance the ionisation of some elements.

FAN concentration, moisture, ash and oil content were determined as reported by Tsouko et al. [22]. Briefly, FAN concentration was assayed by the ninhydrin colorimetric method, while ash and moisture were calculated gravimetrically. Ash was expressed as the percentage of residues left after sample treatment at 550 °C for 4 h and moisture was determined after oven-drying 5 g of the samples to constant weight at 100 °C. The oil content was measured after liquid–liquid solvent extraction using n-hexane (overnight, at 25 °C).

### Statistical analysis

Excel Analysis ToolPak was used for statistical analysis. The data were compared using analysis of variance (ANOVA) and Pearson’s linear correlation at 5% significance level. Significant differences between means were determined by Honest Significant Difference (HSD-Tukey test) at level of *p* < *0.05*. Data were reported as mean values ± standard deviation of three independent replicates (*p* < *0.05*, 95%).

## Results and discussion

### Characterization of glycerol and WDE

**Fig. 1:**
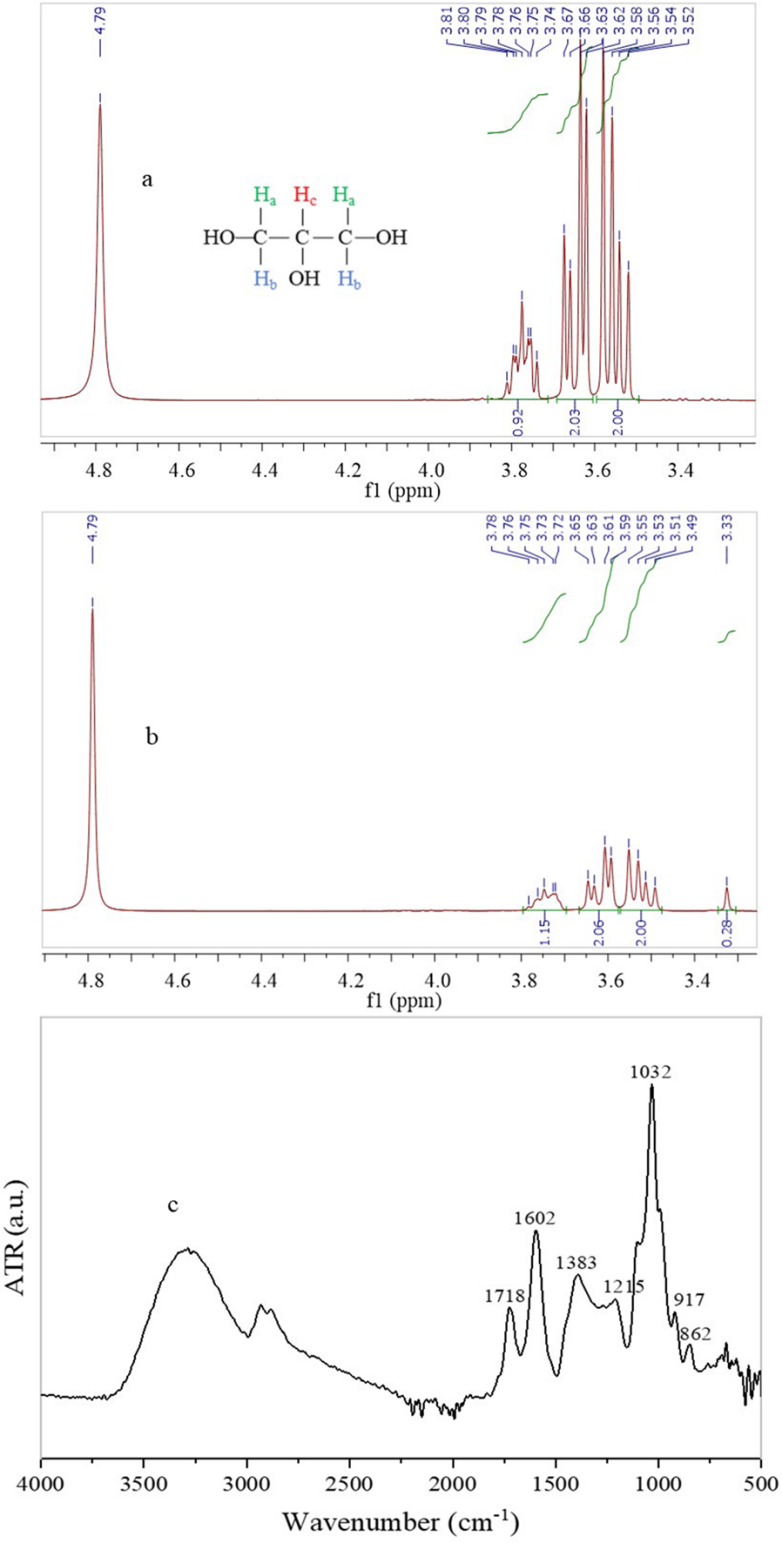
^1^H-NMR spectra for **a** pure glycerol and **b** biodiesel-derived glycerol (crude) in deuterated oxide (D_2_O) (which is observed at 4.79 ppm in the spectra) as well as **c** FTIR spectra of dry WDE

The chemical composition of pure and crude glycerol was monitored via characteristic peaks that appeared in the ^1^H-NMR spectra (Fig. 1a, b). All peaks in the spectra of pure glycerol (Fig. 1a) were shifted by 0.03 ppm compared to the corresponding ones in the crude glycerol spectra. The multiplet between 3.8 and 3.7 ppm can be assigned to the proton (H_c_) attached to the C2. The doublet of doublets between 3.7 and 3.6 ppm and 3.6–3.5 ppm can be assigned to the protons (H_a_ and H_b_) on C1 and C3 [25]. In the case of crude glycerol, the peak at 3.3 ppm was assigned to methanol (Fig. 1b) which derives from the biodiesel production [26]. Signals of other impurities such as potassium and sodium salts that result from catalysts e.g., NaOH and KOH involved in the transesterification process of oils to produce biodiesel, were not observed. High salts concentrations can lead to cell rupture due to osmotic pressure and reduced respiratory activity of cells that is interlinked with deficiencies in cell growth and substrate uptake rates. Strains of acetic acid bacteria are inhibited in the presence of 1–5 g/L NaCl and KCl, in detriment to the BC synthesis [27].

WDE after centrifugation, showed an acidic pH-value of 3.0 ± 0.01, which is in accordance with relevant studies that report pH values within 3.2–4.1 [3, 4, 28, 29]. However, the total solids of WDE were much lower (7.28 ± 0.44 g/L) to those reported by other studies (18.3–29 g/L) [3, 4, 28, 30]. The ash and oil contents determined were equal to 6.10 ± 0.10 g/L and 0.43 ± 0.07 g/L, respectively. The FAN concentration was quiet lower (38.42 ± 0.29 mg/L) compared to the ammonia nitrogen (135 mg/L) reported by Petta et al. [30]. Higher TPC (590–2,500 mg GAE/L) compared to the present study (378.45 ± 36.18 mg GAE/L), have been found by Petta et al. [30] and Salgado et al. [31]. WDE properties are dynamic, mostly affected by the grape variety, soil and the climate. The aforementioned variations are also attributed to the fact that WDE evaluated in this study was obtained after the sedimentation of waste yeasts and suspended solids, while it was further centrifuged prior to characterization. It is noted that after thorough investigation of the literature, there is no publication reporting the individual sugar/carbon composition of WDE. In this study, concentrations of glucose, fructose and glycerol were found to be 5.00 ± 0.31 g/L, 5.30 ± 1.40 g/L and 9.85 ± 0.89 g/L, respectively.

Based on the FTIR spectra of WDE (Fig. 1c), the absorbance band at around 3284 cm^−1^, detected in the high frequency region of 3675–3000 cm^−1^, was attributed to stretching vibration of OH and alcohol groups. Moreover, this region is also typical for the hydrogen bonds of H_2_O [32]. The peaks at 2937 cm^−1^ and 2889 cm^−1^ were assigned to symmetric and asymmetric CH stretching, respectively [33]. The peaks at 1718 cm^−1^ and 1602 cm^−1^ were related to vibrations of C = O bonds. The shoulder found at 1099 cm^−1^ was attributed to alcohol group C–O bonds matching to sugars or to the C–O bonds of acid, ester and ether groups [32]. Stretching of C–OH and C–C-OH bonds in secondary and primary alcohols were, respectively, reflected by the peak centered at 1099 cm^−1^ and the frequency region within 943–1088 cm^−1^ [33]. The peak observed at 670 cm^−1^, 641 cm^−1^, and 626 cm^−1^, indicated the presence of inorganic salts e.g., Cl^−^ or SO_4_^2−^ [34] that have been reported to be present in vinasse derived from the grape distillation process [30].

### Effect of different mixing ratios of WDE on BC production

The fermentation efficiency was initially investigated in terms of various mixing ratios of WDE diluted with tap water (20 g/L initial crude glycerol). Increasing WDE volume led to the gradual increase of BC concentrations (Table 1), strongly indicating that this newly isolated bacterium is tolerant towards phenolic compounds naturally occurring in WDE. The maximum values of BC concentration (8.1 g/L), productivity (0.81 g/L/d), glycerol consumption (98.9%) and FAN consumption (84.3%) were determined when 100% of WDE was applied. Fructose consumption was moderate in all cases (5.0–25.0%) while glucose was adequately catabolized (70.0–82.4%) by the bacterial strain. Glycerol was almost totally depleted (95.0–98.9%) from the fermentation media after 10 days of cultivation.

**Table 1:** Performance of *K. rhaeticus* on fermentation media containing various mixing ratios of WDE with tap water, supplemented with different glycerol concentrations and the effect of media sterilization method on the fermentation efficiency

WDE	Glycerol (g/L)	BC (g/L)	Productivity (g/L/d)	Carbon sources consumption (% w/w)				FAN consumption(% w/w)	WHC (w/w)^1^
WDE (% v/v)				Glucose	Fructose	Glycerol	Total		
Thermal sterilization									
25	Crude, ≈20	3.7 ± 0.25^f^	0.42 ± 0.04^e^	70.0	6.3	91.5	87.9	71.8	86.2 ± 6.89^b^
50	Crude, ≈20	5.1 ± 0.22^e^	0.56 ± 0.03^d^	72.0	5.0	93.4	83.8	78.4	84.3 ± 5.99^c^
75	Crude, ≈20	6.6 ± 0.22^d^	0.67 ± 0.04^c^	82.0	25.0	92.9	81.7	72.2	89.5 ± 6.35^a^
100	Crude, ≈20	8.1 ± 0.45^c^	0.81 ± 0.06^b^	82.4	14.7	98.9	85.5	83.1	82.6 ± 4.75^d^
100	Crude, ≈30	9.0 ± 0.38^b^	0.90 ± 0.05^a^	58.3	33.3	95.2	81.5	84.3	60.1 ± 3.75^f^
100	Crude, ≈45	4.0 ± 0.25^f^	0.45 ± 0.02^e^	31.4	10.0	65.1	57.6	76.2	87.1 ± 5.76^b^
100	Pure, ≈30	9.8 ± 0.42^a^	0.98 ± 0.06^a^	47.3	35.8	84.5	72.6	87.3	56.6 ± 4.01^ g^
0	Crude, ≈30	5.1 ± 0.29^e^	0.46 ± 0.03^e^	–	–	57.6	57.6	52.1	68.4 ± 4.99^e^
Filter sterilization									
100	Crude, ≈30	6.6 ± 0.15^d^	0.66 ± 0.02^c^	100	17.2	60.4	57.4	72.7	86.0 ± 8.69^b^

^1^ g water/g dry BC; Different superscript letters within same individual columns indicate statistically significant differences (*p* < *0.05*)

The production of gluconic acid during fermentation has been reported by many publications [35], nevertheless, in the present study, gluconic acid was detected in negligible concentrations (< 0.7 g/L). In fact, the average pH values in all fermentations fluctuated between 4.8 and 5.3. This could probably be attributed to the phenolic compounds contained in WDE. It has been reported that compounds with antioxidant nature e.g., lignosulphonates and phenolic-rich hydrolysates (e.g., molasses), are inhibitory to gluconic acid production [36]. Another possible explanation is that when glucose is used as a sole carbon source, it serves both as an energy source (via oxidation into gluconic acid) and as a BC precursor. The increased gluconic acid production is to the detriment of BC production as it competes with BC for the carbon source and it also drops the media pH to suboptimal values, thus hampering the bacterial viability [36]. In our study, glycerol appeared to serve as a direct source of energy. Glycerol can turn the pathway from the TCA to the Krebs cycle, employing oxidation of triose phosphate without production of gluconic acid [7]. Therefore, it is assumed that glycerol and glucose were used as BC precursors [37].

After thorough investigation of the literature, the authors did not find any publications dealing with the valorization of wine vinasses (liquid) towards BC production. Very few studies have examined solid winery waste streams. Indicatively, soluble sugars (glucose and fructose) extracted from grape pomace led to the production of 9 g/L of BC with a productivity of 1 g/L/day after 9 days of fermentation [38]. In another study, skins, pulp, seeds and stems contained in grape pomace combined with discharged potatoes (ratio of 50:50 w/w) were thermally treated using acid-assisted hydrolysis. The rich in sugar hydrolysate was suggested as efficient fermentation media to produce 4 g/L BC in 6 days [39].

A few publications investigate the utilization of vinasses deriving upon fermentation of crude sugarcane or beet molasses to produce ethanol, or distillery effluent procured from UB breweries, for BC production. For example, dry weight of BC reached up to 0.28 g/L when *K. xylinus* PTCC 1734 was cultivated in vinasses (derived from the anaerobic fermentation of molasses to produce alcohol), with BC production not significantly affected by the proportion of vinasses (up to 45%) [40], while a quite low WHC of 45% was reported. Another study reported that HS-based media, substituting peptone and yeast extract with beet vinasses, led to increased BC concentration (7.8 g/L) compared to the conventional HS-media when *K. xylinus* DSM 2325 was used [37]. *K. saccharivorans* cultivated in crude distillery effluents from UB breweries resulted in quite moderate BC production of 1.24 g/L after 8 days while a 23.6% decrease of the substrate COD was achieved [41].

Overall, this part of the study provides valuable insights into the potential utilization of winery waste for sustainable biopolymer production suggesting avenues for further research. While this study varied the mixing ratios of WDE, further analysis of the phenolic compounds within the effluent could elucidate the effect of individual components in BC synthesis. Understanding the mechanisms by which WDE influence bacterial growth and cellulose production could guide the development of optimized WDE formulations for enhanced BC yields. Additionally, the scalability and cost-effectiveness of BC production using WDE could offer economic opportunities for wineries seeking to implement sustainable practices and reduce waste disposal costs. The results of this study are promising since WDE can directly be utilized for BC production without water input in the process or phenolics removal/dilution, as the concentration of phenolic compounds in WDE did not inhibit BC production.

### Effect of initial crude glycerol concentration on BC production

**Fig. 2:**
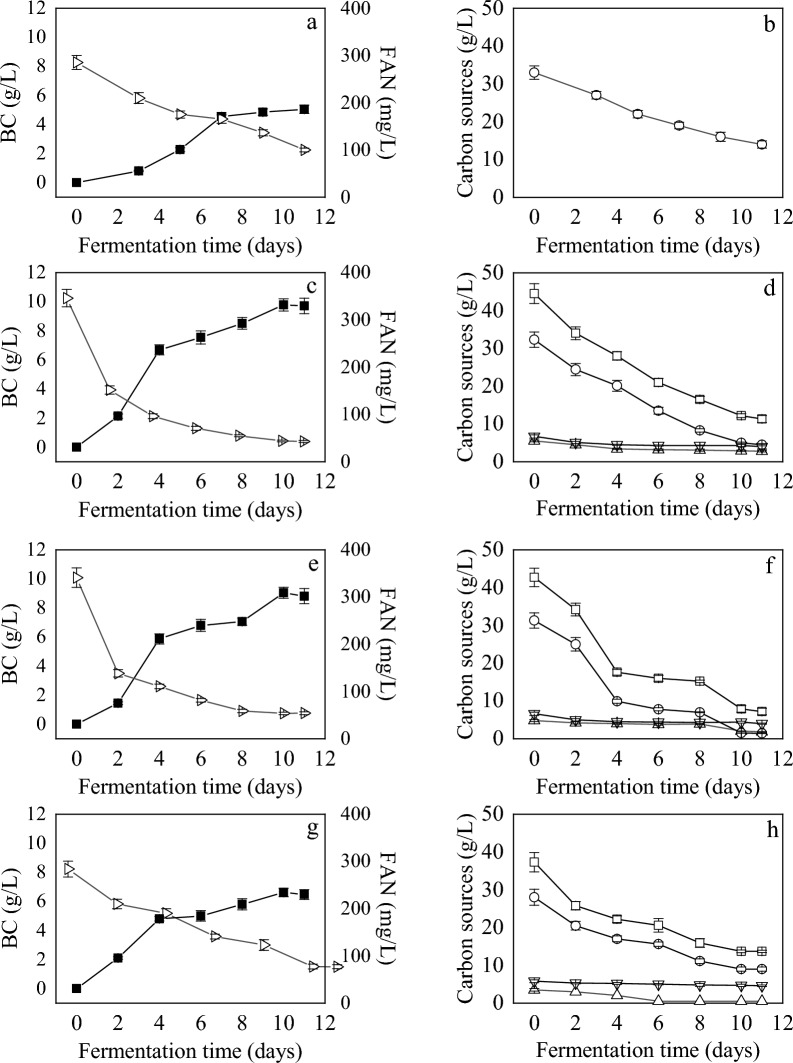
BC production (■), FAN consumption (▷) and carbon sources consumption when fermentations of *K. rhaeticus* were performed using 0% WDE supplemented with 30 g/L crude glycerol (**a**, **b**), 100% WDE supplemented with 30 g/L pure glycerol (**c**, **d**), 100% WDE supplemented with 30 g/L crude glycerol (**e**, **f**), and filter sterilized media of 100% WDE supplemented with 30 g/L crude glycerol (**g**, **h**). Total carbon (□); glycerol (○); glucose (△); fructose (▽)

Subsequent experiments were performed using 100% WDE supplemented with different initial crude glycerol amounts (30 and 45 g/L). When glycerol was increased from 20 g/L to 30 g/L, the fermentation efficiency was significantly enhanced demonstrating the tolerant nature of the bacterium towards osmotic challenges, posed by increasing glycerol concentration (Table 1). FAN (Fig. 2e) and glycerol (Fig. 2f) were steadily consumed within the 10 days of fermentation. The catabolic profile of fructose and glucose (Fig. 2f) was quite slow throughout the fermentation. BC production continuously increased (Fig. 2e), reaching its maximum value of 9.0 g/L after 10 days (Table 1). These very promising results could be due to the presence of hexoses (glucose and fructose) in the fermentation media at a ratio of hexoses to crude glycerol of 1:3 (g/g). It has been previously reported that glucose and glycerol at a ratio of 1:3 reached the maximum BC concentration of 8.4 g/L (productivity of 1.2 g/L/d), while BC production with *Gluconacetobacter xylinus* ATCC53524 decreased (BC = 5.9 g/L) when glycerol or glucose was used as the sole carbon source. The metabolic analysis revealed that the activities of crucial enzymes in BC biosynthesis, namely phosphoglucomutase and UDP-glucose pyrophosphorylase, were around two times higher when glucose and glycerol were added at a ratio of 1:3 in the fermentation media compared to the glucose-based media. The media supplementation with glycerol triggers intermediates of the PPP to enter the TCA cycle and to produce more ATP, and eventually more BC precursors are generated via gluconeogenesis [35].

At this stage of the study, an extra experiment was performed with tap water instead of WDE and 30 g/L crude glycerol (Table 1 and Fig. 2a, b) to validate the positive effect of WDE on BC production by *K. rhaeticus.* The fermentation efficiency decreased in terms of BC concentration (5.1 g/L), productivity (0.46 g/L/d) and glycerol consumption (57.6%), indicating the favorable effect of WDE which can be primarily attributed to the presence of phenolic compounds found in WDE.

Increasing glycerol amount up to 45 g/L, led to a drastic decrease of BC yield and productivity (Table 1). The substrate inhibitory effect due to glycerol excess is a common phenomenon in microbial fermentation. Additionally, high osmotic pressure of the fermentation media caused by e.g., high glycerol concentrations, can dehydrate the microbial cells, hindering their proliferation and the biometabolite’s synthesis [42, 43]. Soemphol et al. [44] reported that the highest BC production was achieved when the newly isolated strain *Gluconaceter xylinus* BNKC19 was cultivated in 10 g/L of initial crude glycerol concentration while increasing crude glycerol up to 50 g/L led eventually to decreased BC (3.1 g/L). The authors stated that this tendency may be due to impurities contained in crude glycerol that could have affected the cells viability. The strain performed also well (8 g/L) when 30% pineapple peel extract was supplemented with 10 g/L of crude glycerol [44]. Yang et al. [27] demonstrated that initial glycerol concentration in the range of 30–50 g/L act inhibitory to BC synthesis with *G. xylinus* KCCM 41431. They reported the best BC concentration of 6.95 g/L with a productivity of 0.99 g/L/d, at 20 g/L initial crude glycerol, 9 g/L yeast extract and 9 g/L peptone. The same trend was reported by Gayathri and Srinikethan (2018), with the highest BC (12.6 g/L) obtained at 20 g/L crude glycerol with *K. saccharivorans*. The effect of initial glycerol amount (10–150 g/L) on BC yield was also investigated by Dikshit and Kim [42], cultivating *G. xylinus* KCCM 41431 both in pure and crude glycerol. Both types of glycerol showed the same tendency at increasing concentrations. More specifically, glycerol concentration higher than 50 g/L (and up to 150 g/L) led to a significant decrease in BC yield, obviously indicating the inhibitory effect of the substrate excess. The production of 1,3-propanediol with *Klebsiella pneumoniae* was inhibited with glycerol concentrations ≥ 60 g/L, with both substrate consumption and biomass formation being decreased. The authors related this performance to difficulties with mass transfer in the media and high osmotic pressure that led to a decrease in cellular uptake, when high glycerol concentrations were employed [46].

### Effect of type of glycerol on BC production

The purity of glycerol may cause a negative impact on the cells’ growth and on the synthesis of the biopolymer [26]. Therefore, in subsequent experiments, 100% WDE was supplemented with pure glycerol (30 g/L), instead of the crude type (Table 1 and Fig. 2c, d). In this case, consumption of glucose (47.3%), and glycerol (84.5%) was lower compared to the crude glycerol while fructose (35.8%) and FAN (87.3%) consumption was slightly higher. The consumption pattern of the glucose, fructose (Fig. 2d) and FAN (Fig. 2c) was quite similar to the fermentation performed with crude glycerol (Fig. 2f). The absence of inhibitory compounds in pure glycerol led to higher uptake rates, especially from day 4 to day 10 (Fig. 2d) compared to crude glycerol (Fig. 2f). The increase of BC concentration was just 8.9% (Table 1), indicating that this newly isolated strain is quite tolerant towards impurities such as sodium and potassium salts found in crude glycerol. These results are in accordance with Dikshit and Kim [42] that reported a 16% increase on BC yield (from 2.93 g/L to 3.4 g/L) when 50 g/L pure glycerol was used, compared to the crude type after process optimization. In another study, the novel strain *Komagataeibacter* sp. nov. CGMCC 17276, was able to produce BC (around 6 g/L in optimized media) without significant differences when it was grown on 50 g/L waste glycerol (WG2) and acetic acid–ethanol-based medium (AEM), compared to pure glycerol and AEM [47].

The choice between pure glycerol and crude glycerol for BC production can have significant implications in terms of cost-effectiveness, scalability, and environmental sustainability. Pure glycerol typically undergoes extensive purification processes to remove impurities, resulting in higher production costs compared to crude glycerol [48]. However, the use of pure glycerol can lead to more consistent BC production outcomes due to the absence of impurities that may affect bacterial growth and cellulose synthesis. Despite this, in this study, efficient BC production was observed when crude glycerol was used instead of pure glycerol, with BC concentration showing a decrease of just 8.2%. To add, scaling up BC production using pure glycerol may pose challenges in sourcing sufficient quantities at a reasonable cost in contradiction to crude glycerol that is readily available in large quantities from biodiesel refineries, making it a more scalable option . Finally, the valorization of pure glycerol may lead to greenhouse gas emissions and chemical waste generation due to energy-intensive purification processes. On the other hand, converting low-value crude glycerol into valuable products addressed broader goals of sustainability and resource efficiency [49].

### Effect of sterilization method on BC production

WDE has been reported to contain polyphenols (e.g., tannins), quite low protein content and several anionic or cationic forms of metals [29–31]. In this study, and up to this step, the preparation of the fermentation media to obtain sterile conditions, was performed via the heat sterilization of WDE, creating coagulation and/or flocculation [50] (optical observation). This phenomenon can be attributed to several reasons. For instance, coagulation could have been created by the impact of the high temperature (121 °C) on some of the aforementioned WDE compounds, forming hydrolyzable metal cations e.g., aluminum sulfate, ferric chloride, ferrous sulfate etc. Also, flocculation could be attributed to the presence of tannins in WDE. Tannins have been proposed as natural flocculants that destabilize organic or/and inorganic particles from wastewater via polymers or hydrogen bridging [50]. To add, elevated temperatures can lead to the polymerisation of phenolic compounds found in WDE, which leads to the formation of brown-coloured and quite turbid fermentation media, as observed in the present study. Manzocco et al. [51] found that heat promotes the polymerisation process of individual phenolic compounds, oligomeric or polymeric tannins. They reported that pasteurization of green and black tea extracts increased the optical density of the beverages [51]. Flocculent precipitation has been also reported in fermented jujube juice due to high-temperature treatment [52]. Flocculation could also has been formed by the change in the solubility of proteins (decreased) or heat coagulation of proteins due to the effect of calcium ions under heat treatment for 15 min at 120 °C, as suggested by de Wit and Klarenbeek [53]. Some synergies of all the aforementioned could be the actual reason for the creation of these coagulants/flocculants in the present study.

To monitor the effect of coagulants/flocculants on BC production, filter sterilized media was applied in sequential fermentations (Table 1 and Fig. 2g, h). Based on visual observations, the coagulants and/or flocculants formed after the thermal treatment of WDE, had a low colloidal stability since after 4 days of fermentation they were very loosely attached as aggregates on the bottom part of the produced BC membrane. This random but repeated (in all fermentations) media conformation appeared to favor BC production compared to ultra clear media derived after filter sterilization. Also, it was observed that thermally treated media led to the formation of a quite thick and compact BC pellicle immediately after 24 h of fermentation while BC produced in membrane-sterilized media was quite thin after 3 d of fermentation. Based on the aforementioned, it could be hypothesized that coagulants and/or flocculants may have promoted the bacteria immobilization, accelerating the BC biosynthesis. This phenomenon is in accordance with the study of Saleh et al. [54] and Tian et al. [55]. More specifically, in the study of Tian et al. [55], the supplementation of fermentation media with 2% mesoporous halloysite nanotubes (HNTs), enhanced the efficiency of *G. xylinus* with BC being increased by 2.7-fold (5.9 g/L) after 15 days of static incubation. HNTs seemed to act both as a cell immobilization agent and oxygen source while insignificant HNTs residues were found in the BC membrane after purification.

### Properties evaluation of the produced BC

**Fig. 3:**
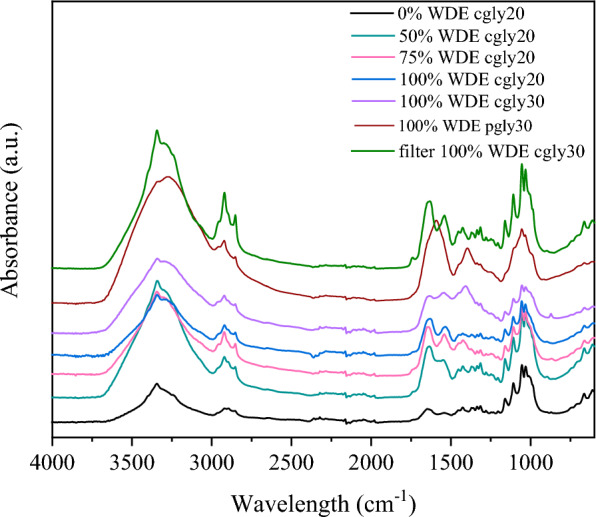
FT-IR spectra of BC samples produced by *K. rhaeticus* utilising WDE (0–100%) and glycerol (crude and pure) in thermally- and filter-sterilized fermentation media

The functional groups of BC samples were monitored using FT-IR analysis (Fig. 3). The profile of the FT-IR spectra was similar for all BC samples, with typical cellulose vibration bands and in accordance with previously available literature reports related to this study [18, 20]. The peak at 3347 cm^−1^ was attributed to –OH stretching vibrations. CH stretching vibrations of BC, including –CH_2_ and -CH_3_, were recorded at 2913 cm^−1^ and 2856 cm^−1^, respectively. The peak at around 1159 cm^−1^ belongs to C–O–C group of the polysaccharide skeleton, while peaks at 1106 cm^−1^, 1056 cm^1^ and 1029 cm^−1^ represent C–OH bonds of BC. The peak at around 1621 cm^−1^ is attributed to -OH bending.

**Fig. 4:**
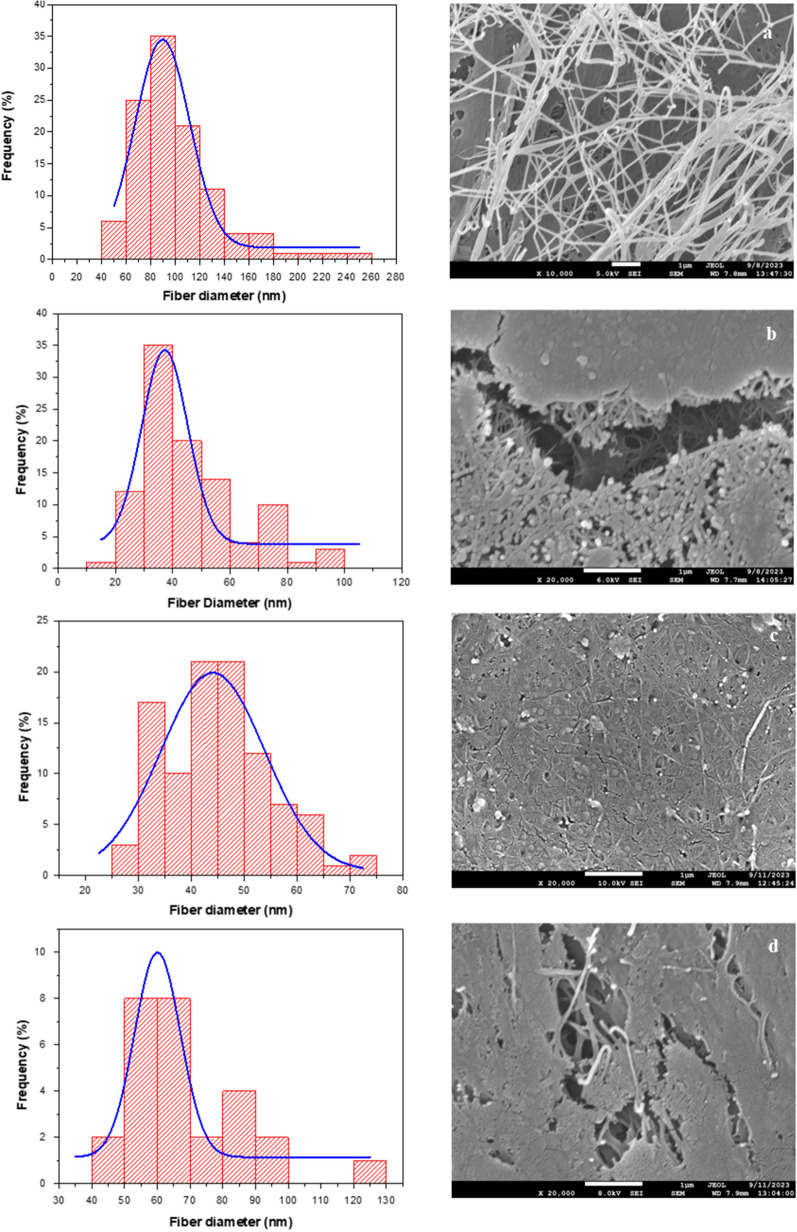
SEM micrographs of BC produced by *K. rhaeticus* using **a** filter-sterilized media: 100% WDE + 30 g/L crude glycerol; **b** heat-sterilized media: 100% WDE + 30 g/L crude glycerol; **c** heat-sterilized media: 0% WDE + 30 g/L crude glycerol and d) heat-sterilized media: 100% WDE + 30 g/L pure glycerol. (**a** × 10,000 magnification; **b**, **c**, **d** × 20,000 magnification). Diagrams present the respective fiber diameter of BC samples as aforementioned

SEM micrographs of BC samples produced by *K. rhaeticus* using different experimental conditions are presented in Fig. 4. BC from filter-sterilized 100% WDE supplemented with crude glycerol showed a continuous fibrillar three-dimensional network of high porosity (Fig. 4a) that can be related to its high WHC (86 g water/g dry BC). The fibers’ diameter ranged from 10 to 100 nm while the average fiber diameter was calculated to be 89.62 ± 32.04 nm. When the fermentation media was heat-sterilized, the morphology of BC regarding the inner matrix was characterized by a denser fibril network, whilst the average diameter values were within 37.20 ± 18.99 nm (Fig. 4b). Interestingly, the outer surface core seemed to have amorphous regions combined with complexes of phenolic compounds and BC fibrils despite the thorough washing of BC. BC produced when 0% WDE and crude glycerol were used (Fig. 4c) showed an extremely dense and almost compact morphology, forming domains of low porosity, with a tendency to form agglomerates. The average fiber diameter was 44.07 ± 19.13 nm. BC derived from 100% WDE supplemented with pure glycerol presented an amorphous-like core surface area while the inner BC matrix was dense and quite compact with an average fiber diameter of 60.03 ± 16.51 nm (Fig. 4d).

The composition of the fermentation media has been reported to affect the surface morphology and the fibril width of BC. For example, higher fibrillar variations (27 ± 10 nm) were observed in SEM micrographs of BC produced using crude glycerol combined with sunflower meal enzymatic hydrolysates as the fermentation media compared to that derived from the HS medium (34 ± 2 nm). To add, these BC membranes showed a more disorganized network, which consisted of random overlaying fibrillar domains compared to BC produced from HS medium in which the carbon source was substituted with glycerol [56]. In another study, BC produced from enzymatic hydrolysates of wheat milling by-products was characterized by an organized and continuous network with fibril diameters ranging from 45.8 to 73.1 nm [57]. Andritsou et al. [58] reported that BC produced from orange peel aqueous extract presented a long fibrous network of microfibrils with diameter values within 50–100 nm. Selianitis et al. [59] have provided a very thorough review of nanocellulose production from numerous lignocellulosic feedstock. Indicatively, nanocellulose extraction from flax, sisal and hemp was carried out using H_2_SO_4_-assisted hydrolysis to obtain structures of respective fibrillar diameters of 15–45 nm, 10 nm, and 20–50 nm. In the case of ultrasonic-assisted extraction, the nanocellulose derived from wood, bamboo, wheat straw and flax presented fiber diameters of 10–20 nm, 10–40 nm, 15–35 nm and 15–100 nm, respectively. The enzymatic hydrolysis of lignocellulosic biomass (with the synergistic action of cellulases and xylanases) results in the production of cellulose structured in bundle of fibrils with diameter values in the range of 5–110 nm.

### Evaluation of phenolics adsorption on BC and dephenolization

**Fig. 5:**
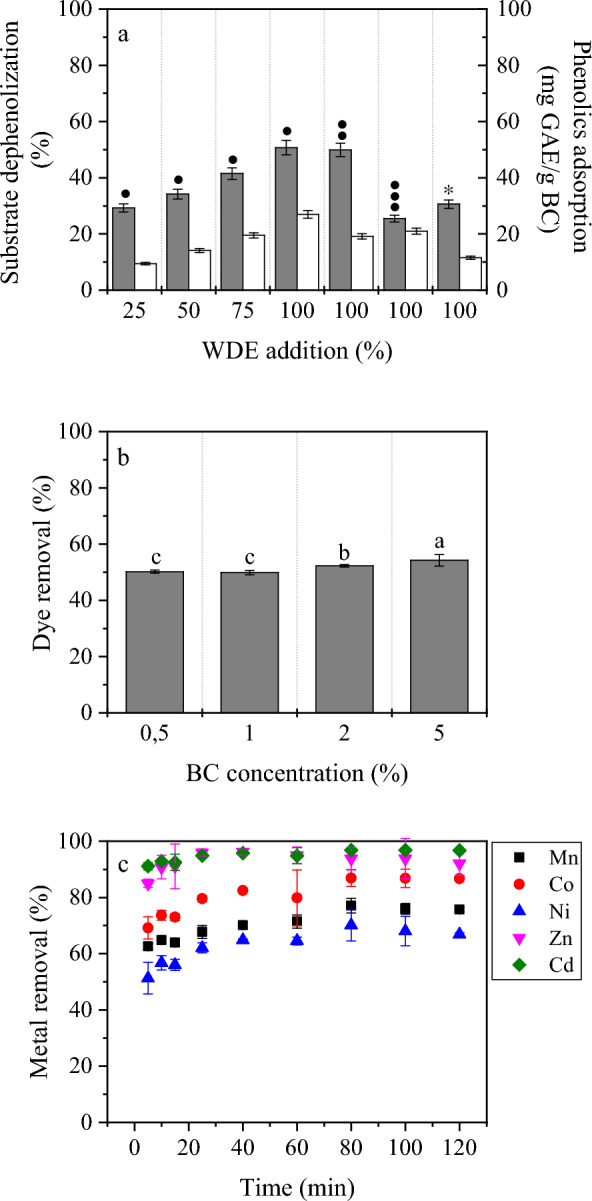
**a** Dephenolization potential (grey bars) and phenolics adsorption (white bars) of BC membranes at the end of each fermentation containing different concentrations of WDE (from 25 to 100%) in fermentation feedstock supplemented with crude glycerol: 20 g/L (●), 30 g/L(●●), 45 g/L (●●●), and 30 g/L pure glycerol (*); **b** removal (%) of the dye acid yellow 17 from synthetic wastewater (initial concentration of 20 mg/L) after 30 min of stirring with different amounts of BC and **c** removal (%) for Mn, Co, Ni, Zn and Cd from synthetic wastewater after 120 min of stirring with 1% BC

Figure 5a depicts the substrate dephenolization and the phenolics adsorption capacity of BC membranes at the end of each fermentation. A gradual increase in the substrate dephenolization and the adsorption capacity of phenolic compounds on the produced BC membranes was observed with increasing WDE levels, from 25 to 100% using 20 g/L crude glycerol. The maximum respective values of 50.7% and 26.96 mg GAE/g dry BC, were achieved by the BC produced on 100% WDE. Phenolics can undergo physical adsorption through non-covalent interactions such as Van der Waals forces, and H-bonding due to the porous structure and large surface area of BC that provides ample sites for phenolics to be adsorbed. BC produced from *G. xylinus* ATCC 53524 has been reported to effectively bind up to 0.6 mg of polyphenols/g BC with a maximal binding capacity up to 1.4 mg of polyphenols/g cellulose, based on Langmuir isotherms predictions [60]. It has been well demonstrated that water content of BC is positively correlated with the adsorption of polyphenols due to availability of free hydroxyl groups found in BC for polyphenol binding [21]. In this set of experiments, this phenomenon was not related to WHC of the BC membranes since the values of the latter (80.6–89.5 g water/g dry BC) (Table 1) showed slight variations without any specific trend. The increasing tendency could be related to the increasing BC concentrations and thus more available area for initially binding onto sites of BC ribbons surface and subsequently, diffusion into the accessible microfibrillar area. The large number of accessible surface sites on BC facilitates multilayer adsorption of phenolic compounds via pore-filling adsorption including hydrophobic interactions and hydrogen bonding [21]. Significant substrate dephenolization (49.9%) was also observed when the initial crude glycerol was increased to 30 g/L while the adsorption capacity of BC decreased (19.2 mg GAE/g dry BC). This can be directly related to the sharp drop in the WHC of the BC membrane (60 g water/dry BC) (Table 1). In the highest crude glycerol concentration (45 g/L) or when pure glycerol was used, the substrate dephenolization decreased to 25.5% and 30.6%, respectively. In wastewater containing multiple contaminants, competitive adsorption phenomena may occur, where different pollutants compete for adsorption sites on BC surfaces. The presence of other organic or inorganic species in the fermentation media due to crude and pure glycerol involvement can influence the adsorption behavior of phenolic compounds. However, despite the much lower BC concentration (4 g/L) when 45 g/L crude glycerol was employed, the BC capacity to bind phenolic compounds was very comparable (21.0 mg GAE/g dry BC) with BC produced on 30 g/L crude glycerol (19.2 mg GAE/g dry BC) probably due to the quite high WHC (87.1 g water/g dry BC). The BC produced using pure glycerol showed moderate substrate dephenolization (30.6%) and phenolics adsorption capacity (11.5 mg GAE/g dry BC) as well as quite low WHC (56.6 g water/g dry BC).

To conclude, understanding the competitive interactions within contaminants contained in wastewater is essential for predicting and optimizing the performance of BC-based adsorbents in complex wastewater matrices that are rich in phenolic compounds. Also, the in-situ modification of BC could lead to tailored properties such as larger-pore structure that allow for easier diffusion of phenolics molecules into the BC matrix.

Fruits and vegetables, besides from fresh consumption, are also processed via freeze-drying, thermal treatment, and juicing. Food processing conditions modify the plants cell wall, leading normally to reduced polyphenols binding affinity and thus reduced bio-accessibility/availability. Another major problem is that manufacturing of raw materials that are rich in phenolic compounds e.g., grapes and fruits/vegetables can adversely affect organoleptic and physicochemical properties of wine and juices [60]. This preliminary in situ approach on the phenols adsorption capacity of BC can give insight and serve as a basis to studies that investigate the effect of processing conditions on the binding mechanism within polyphenols and cell walls of plants, which is rather challenging due to the complex nature of the latter. BC membranes show a tremendous interest as a mimicking model-alternative due to chemical and structural similarities with plant-based cellulose. Most importantly, so far studies are limited to fresh fruits and vegetables models [21, 60]. To add, this study can lead to scientific innovation providing some basic evidence on the designation of safe, green, low cost, and biocompatible nano-sensors based on polyphenol-loaded BC, that allow the visual detection of the spoilage of perishable foodstuff. More specifically, Mohseni-Shahri et al. [61] prepared pH indicators by incorporating anthocyanin and curcumin mixtures on BC nanofiber sheets. The performance of the pH indicator film was investigated by their exposure to fish meat placed in transparent packages. Distinct color changes of the pH indicator were correlated to pH changes of fish meat leading to specific conclusions about the quality of the fish meat. So far, pH indicator films have been prepared using complexes of black chokeberry pomace extract immobilized in chitosan [62], cassava starch and blueberry residue [63] and artemisia gum/red cabbage anthocyanins anchored by carboxymethyl cellulose sodium [64].

### Evaluation of BC for dye removal

Acid yellow 17 is a very important commercial azo dye that is used in the textiles industry, typically on acrylics, wool and cotton blends, while it is also used as a food colourant. Typical concentrations of dyes i.e., in textile effluents varies from 10 to 250 mg/L [65]. Amongst the process that can be used for removal and degradation of acid yellow 17 are Fenton oxidation [66], photo-catalytic degradation [67], microbial biodegradation [68] and eco-friendly bio-absorbents [69]. In this study, the BC potential as a biosorbent for acid yellow 17 removal from wastewater was investigated. According to the results obtained, BC was able to remove more than 50% of 20 mg/L acid yellow 17 aqueous solution under the conditions tested, as illustrated in Fig. 5b. Increased amounts of BC from 1 to 5% showed significant differences on dye removal potential. Dye removal percentages reached up to 49.9% and 52.3% for BC concentrations of 1% and 2%, respectively. The highest removal percentage reached up to 54.3%, which was achieved at a BC concentration of 5%. However, the highest adsorption capacity achieved was 2.01 mg/g BC when 0.5 wt.% BC was used, indicating that dye removal can be studied at a lower BC concentration to achieve the highest adsorption capacity, between other factors e.g., pH, initial dye concentration, etc. [9].

There is a limited number of studies that have investigated the dye adsorption capacity of BC and BC-based composites. BC in the form of spheres produced by *Gluconoacetobacter hansenii* NCIM 2529 has been previously tested for its ability to remove the azo dye Aniline blue. A maximum dye removal of 80% was achieved for an Aniline blue solution of 400 ppm, after 60 min and at pH of 7.0 [70]. Another study tested the removal of Crystal violet and Congo Red dyes using BC pellicles produced from *Acetobacter xylinum* TISTR 975, at a dye concentration of 100 mg/L. Maximum removal of Crystal violet using wet and dry BC was 62.7% and 71.3%, respectively, while respective removal percentages for Congo Red were 44.3% and 35.2%, after 210 min of incubation [71]. The dye removal potential of BC aerogels (produced by *A. xylinum* using pineapple peel waste), was evaluated against several cationic dyes such as Methylene blue, rhodamine B, crystal violet, and malachite green. The respective adsorption capacities were 29.7 mg/g, 28.0, 33.0, and 48.6 mg/g, attributed to the strong interaction between oppositely charged dyes (positive charge) and cellulose aerogels (negative charge). In contrast, anionic dyes, e.g., Methyl orange, Sunset yellow, and Quinoline yellow, were repulsed by the negatively charged BC aerogel [9]. Oxidized BC nanofibrils, blended with a magnetic attapulgite/chitosan composite showed very high adsorption capability (230 mg/g) for the dye Congo Red [72]. In another study, hybrid aerogels of BC (produced by *K. xylinus* ATCC23760) and molybdenum disulfide revealed a 96% removal of methylene blue within 120 min and characterized by a first-order kinetic constant (*K*_*obs*_) of 0.0267 min^−1^, compared to 0.005 min^−1^ achieved by BC alone [73].

### Potential of BC for metal removal

BC was very effective at metal removal from a multi-metal synthetic wastewater (Fig. 5c), showing maximum removal percentages of 77 ± 2% for Mn, 87 ± 0.5% for Co, 70 ± 5% for Ni, 96 ± 1% for Zn and 97 ± 0.5% for Cd. Most of the removal occurred after 40 min, with the sorption reaction reaching an equilibrium. These results highlight the potential application of BC to remediate metals from aqueous media. According to the Environmental Protection Agency, metals such as Pb, Zn, Mn, Cd, Hg and Ag are crucial water pollutants present in effluents of industries such as metallurgy, pharmaceutical, as well as chemical and petroleum refining industries [74]. BC could be used either in the form of spheres with higher surface area and pore volume, or in the form of membranes for membrane filtration technologies.

To the best of the authors’ knowledge, this is the first study that has reported the potential of BC to remove metals in a solution resembling a multi-metal synthetic wastewater. A few previously published studies have explored the potential of BC and BC composites to remove metals from aqueous solutions. For example, the potential of BC membranes to remove Ni^2+^ and Pb^2+^ reached up to 32.1% and 65.9%, respectively, at pH 4, with an initial metal concentration of 5 mg/L and 400 mg/L BC. It was suggested that Ni^2+^ removal is ruled by crystallization on the metals absorbed on the BC active groups, while Pb^2+^ removal is driven by the adsorption process, as indicated by the transmission electron microscopy performed on the spent material [75]. In another study, BC loaded with nano zero valent iron could remove up to 99.96% of trace Cd^2+^ from water, present at an initial concentration of 10 mg/L, under a broad pH range of 5–8 [76]. Further research is required to determine sorption kinetics, desorption, metal recovery and/or reusability of BC. To optimize metal removal and recovery, and achieve the highest metals adsorption capacities, pH, temperature and ionic strength of the solution, as well as metal concentration and adsorbent dosage need to be studied.

## Conclusions

In this work, low-cost feedstock from wine distilleries and the biodiesel industry were used to produce BC in high amounts with *K. rhaeticus*. The presence of phenolic compounds in WDE favoured BC synthesis. Crude glycerol (30 g/L) blended with 100% WDE led to the highest fermentation efficiency (9 g/L). The formation of coagulants and/or flocculants after thermal media sterilization created a media conformation that may have promoted the bacteria immobilization, enhancing the BC production. The absorbance experiments revealed the high capacity of the BC membrane to remove highly toxic for the terrestrial and aquatic territories compounds including phenolics, dyes, and heavy metals. Therefore, the current study provided a circular-oriented alternative to produce sustainable biopolymers with promising application in wastewater treatment applications. Future research should focus on BC functionalization/modification to develop BC-based adsorbents that can (a) capture valuable organic compounds from wastewater, which can be further recovered via regeneration, thus addressing resource recovery and (b) complement existing wastewater treatment processes by targeting specific contaminants that are challenging to be removed via conventional practices, thereby achieving higher overall treatment efficiencies.

## Data Availability

All data generated or analyzed during this study are included in this published article.

## Abbreviations

UDP-Glc: Uridine diphosphoglucose
PPP: Pentose phosphate pathway
TCA: Tricarboxylic acid cycle
BC: Bacterial cellulose
WDE: Wine distillery effluent
WHC: Water holding capacity
FAN: Free amino nitrogen
SEM: Scanning electron microscope
ATR-FTIR: Attenuated total reflectance Fourier Transform infrared spectroscopy
TPC: Total phenolic content
GAE: Gallic acid equivalent
HPLC: High-performance liquid chromatography
HNTs: Halloysite nanotubes
